# Complication Rate of the Nuss Procedure in Adults and Pediatric Patients: National Database Analysis

**DOI:** 10.1016/j.atssr.2024.04.013

**Published:** 2024-04-27

**Authors:** Hamza Rshaidat, Eliyahu Gorgov, Micaela L. Collins, Shale J. Mack, Gregory L. Whitehorn, Jonathan Martin, Luke Meredith, Avinoam Nevler, Olugbenga T. Okusanya

**Affiliations:** 1 Division of Esophageal and Thoracic Surgery, Thomas Jefferson University Hospital, Philadelphia, Philadelphia; 2 Division of Hepatobiliary and Pancreatic Surgery, Department of Surgery, Thomas Jefferson University Hospital, Philadelphia, Pennsylvania; 3 Sidney Kimmel Medical College, Thomas Jefferson University, Philadelphia, Philadelphia

## Abstract

**Background:**

Pectus excavatum (PE) is the most common congenital chest wall defect and is characterized by the inward displacement of the sternum and costal cartilages. To date, there are limited data on adult patients undergoing the Nuss procedure for PE. This study aimed to assess the complication rate between the pediatric and adult populations and assess the trends in demographics.

**Methods:**

Retrospective analysis was conducted using a global health care database, TriNetX. Current Procedural Terminology codes (21742, 21743) were used to identify all patients who underwent Nuss procedures in the years 2004 to 2023. The cohort was then subdivided on the basis of age and sex. These patients were assessed for 30-day and 90-day major and minor postoperative complications, as well as acute pain and chronic postoperative pain.

**Results:**

A total of 2843 patients who underwent Nuss repair were identified. Patients aged >18 years had increased hemorrhagic complications (3% vs 0.86% in patients aged <18 years; *P* < .001) and acute pain (55% in patients aged >18 years vs 39.1% in patients aged <18 years; *P* < .001). Overall complication rates were 28.48% in female patients and 21.7% in male patients (*P* = .0014). Female patients had higher rates of respiratory complications (6% vs 2.7% in male patients; *P* = .001), chronic pain (5.2% in female patients vs 2% in male patients; *P* < .001), and hemorrhagic complications (6% in female patients vs 0.97% in male patients; *P* = .0042).

**Conclusions:**

This study suggests that adults with PE experience significantly increased postoperative pain and hemorrhagic complications after the Nuss procedure when compared with the pediatric population. Female patients experience significantly higher complication rates when compared with male patients in all age groups.

## Visual Abstract

**Figure:**
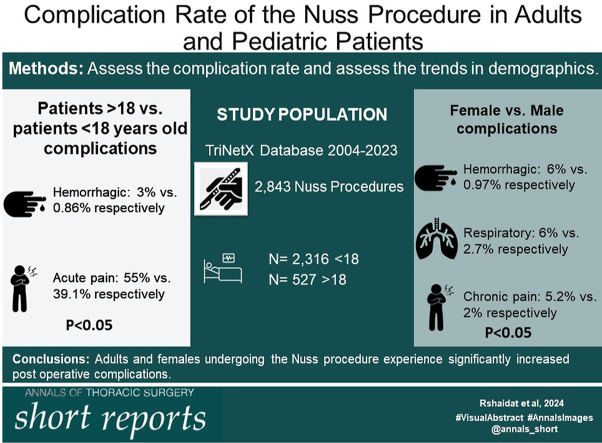

**In Short**

▪ Adults experience significantly increased complications compared with the pediatric population after undergoing the Nuss procedure for pectus excavatum repair.
▪ Female patients experience higher complication rates regardless of age.

Pectus excavatum (PE) is the most common congenital chest wall defect and is characterized by the inward displacement of the sternum and costal cartilages. PE affects 1 in 400 births, with a 3:1 male predominance.^1^ Historically, the Ravitch procedure has been the mainstay treatment for PE. The technique was first described in 1949 and involved perichondrial resection of all deformed costal cartilages, sternal osteotomy, and fixation using an implantable bar.^1^ In 1998, Nuss and colleagues^2^ described a minimally invasive approach to PE repair. The repair consisted of thoracoscopically guided retrosternal bar placement and stabilization to elevate the sternum upward.

The Nuss procedure is now a widely accepted treatment choice for PE. To date, most studies describing increased complication rates in adults are single-institution studies.^3^ Studies using national representative data observed higher complication rates in adults when compared with the pediatric population.^4^ In contrast, other studies observed comparable rates in postoperative complications in both populations.^5^

Given the rarity of the condition, there are limited national data on the procedure and its true complication rate. In this study, we aimed to describe the complication rate in patients undergoing the Nuss procedure for PE by using a global health care database. The study focused on describing demographic trends and the distribution of procedures performed within the TriNetX database.

## Material and Methods

### Study Design

This large, retrospective cohort study was conducted using the TriNetX research network. TriNetX is a global federated health research network with access to deidentified data from electronic health records across different health care organizations worldwide. Network details of previous studies have been described extensively.^6^

### Patient Identification

Patients were identified using operative codes from coding classification of Current Procedural Terminology (CPT). The study included patients who underwent the Nuss procedure from 105 health care organizations using CPT codes (21742, 21743). For analysis purposes, the cohort was then subdivided on the basis of sex for the first analysis; for the second analysis, the subdivision was based on age, dividing the pediatric population from the adult population.

### Outcome

Outcome were defined according to the following classification systems: standard International Classification of Disease 10th Revision Clinical Modification (ICD-10-CM) for medical diagnoses; International Classification of Disease, Ninth Revision Clinical Modification (ICD-9-CM) subclassification and CPT for operative codes; Logical Observation Identifiers Names and Codes (LOINC) system for laboratory analysis; Veterans Affairs (VA) system, RxNorm, and Anatomical Therapeutic Chemical (ATC) system for medication used postoperatively. The associated risk for each outcome was measured between the groups for 30 and 90 postprocedural days. All the codes used for patients’ identification and outcome analysis are shown in Supplemental Table 1. Complications were subclassified as major or minor at the authors’ discretion (Supplemental Table 2). The Clavien-Dindo classification, the Comprehensive Complication Index, or the Accordion Severity Classification could not be used during our analysis because the TriNetX database lacks data on the treatment of specific complications.

### Statistical Analysis

The statistical analysis was performed within the TriNetX platform. The occurrence of outcome was measured, with analysis of risk difference, risk ratio, odds ratio, probability of the outcome at a respective time interval (Kaplan-Meier curve), log-rank test, hazard ratio, and test for proportionality between the 2 groups. Statistical significance was defined as *P* <.05.

## Results

A total of 2843 patients who underwent Nuss repair were identified within the database. All procedures identified were procedures performed in the Unites States, and there were no data available for procedures performed outside the United States. The mean age of patients aged <18 years was 14.6 years compared with 22.1 years for patients aged >18 years. Most of the patients (2316; 81%) were aged <18 years, and 1826 (86%) of this group were male. There were 527 patients aged >18 years, and 366 (74%) of them were male (Table 1).

**Table 1:** Demographics

Characteristic	Full Cohort	<18 Years	>18 Years	*P* Value
Patients, n	2843	2316	527	…
Age, y		14.6 ± 1.93	22.1 ± 6.38	<.001
Gender				
Male		1,826 (86)	366 (74)	<.001
Female		307 (14)	128 (26)	<.001
Race				
White		1856 (80)	453 (86)	.0020
Unknown		53 (10)	364 (16)	.0009
Black		29 (1)	10 (or less)	…
Asian		34 (2)	10 (or less)	…
AI/AN		0	0	…
NH/PI		10 (or less)	10 (or less)	…
Hispanic		206 (9)	45 (9)	.7950
Marfan syndrome		64 (3)	20 (4)	.2068

Values are n, mean ± SD, or n (%).

AI/AN, American Indian/Alaska Native; NH/PI, Native Hawaiian/Pacific Islander.

There was no significant difference in overall complications, minor complications, or major complications between both age groups. However, when looking at individual complications, patients aged >18 years had increased hematologic complications (3% as opposed to 0.86% in patients aged <18 years; *P* < .001). Additionally, patients aged >18 years had increased acute postoperative pain (55% vs 39.1% in patients aged <18 years; *P* < .001). There were no other significant differences in other complication categories (Table 2).

**Table 2:** Complications Classified by Age

Complication	<18 Years, n (%)	>18 Years, n (%)	*P* Value	OR	95% CI
Any complication	521 (22.5)	129 (24.5)	.3280	1.117	0.895-1.393
Major complication	97 (4.2)	31 (5.9)	.0905	1.43	0.943-2.167
Minor complication	461 (19.9)	108 (20.5)	.7606	1.037	0.82-1.312
Respiratory	68 (2.9)	24 (4.6)	.0582	1.577	0.981-2.537
Hematologic	2 0 (0.86)	16 (3)	<.001	3.595	1.85-6.985
Thrombotic	0 (0)	0 (0)			
General postoperative	444 (19.2)	97 (18.4)	.6864	0.951	0.746-1.213
Acute pain	905 (39.1)	290 (55)	<.001	1.908	1.576-2.309
Chronic pain (>90 d)	52 (2.3)	17 (3.4)	.1356	1.523	0.873-2.657
Infectious	38 (1.6)	10 or less	.6796	1.16	0.574-2.342

OR, odds ratio.

Female patients had a higher rate of overall complications (28.48% compared with 21.7% in male patients; *P* = .0014). The median age of female patients was 23.1 years, and it was 21.8 years for male patients (*P* < .001). Female patients had a higher rate of major and minor complications: 6.2% in female patients vs 4.1% in male patients (*P* = .0426) and 25.7% in female patients vs 18.9% in male patients (*P* < .001), respectively. When looking at individual complications, female patients had higher rates of respiratory complications: 6% vs 2.7% in male patients (*P* < .001); chronic pain: 5.2% in female patients vs 2% in male patients (P < .001); and hemorrhagic complications: 6% in female patients vs 0.97% in male patients (*P* = .0042) (Table 3).

**Table 3:** Complications Classified by Sex

Complication	Male, n (%)	Female, n (%)	*P* Value	OR	95% CI
Any complication	514 (21.7)	133 (28.48)	.0014	1.437	1.149-1.797
Major complication	97 (4.1)	29 (6.2)	.0426	1.551	1.012-2.377
Minor complication	447 (18.9)	120 (25.7)	<.001	1.487	1.179-1.875
Respiratory	63 (2.7)	28 (6)	<.001	2.335	1.479-3.686
Hematologic	23 (0.97)	12 (2.6)	.0042	2.69	1.329-5.445
Thrombotic	0 (0)	0 (0)			
Acute pain	977 (41.2)	216 (46.3)	.0450	1.226	1.004-1.497
Chronic pain (>90 d)	46 (2)	23 (5.2)	<.001	2.72	1.631-4.535
Infectious	40 (1.7)	10 (or less)	.4967	1.274	0.633-2.566
General postoperative	433 (18.3)	106 (22.7)	.0261	1.313	1.032-1.669

OR, odds ratio.

## Comment

This is a large retrospective study sample using a national database. We observed a higher number of male patients who underwent the Nuss procedure regardless of age. Compared with the pediatric population, we observed higher complication rates in the adults who underwent the Nuss procedure. Additionally, female patients experienced higher complication rates regardless of age. With regard to pain, both adults and female patients had higher rates of postoperative pain when compared with their respective groups.

We believe that the larger number of male patients observed in our cohort reflected the higher male prevalence of PE in a population with a 3:1 male-to-female predominance.^1^ We were not able to extrapolate the indication for surgery or the severity of symptoms between male and female patients from our data. It is possible that male patients present with a higher Haller index, which is a measurement that indicates the severity of the deformity. Additionally, it is possible that clinically asymptomatic girls and women experience less psychosocial distress because of the masking effects of their breast tissue, thus making it less likely for them to seek surgical interventions. Furthermore, women could undergo cosmetic procedures, such as the insertion of breast implants or fat grafting, to mask their deformity.^7^ Further studies should be performed to assess this trend.

Evidence from single institutions has shown increased postoperative complications in adults when compared with the pediatric population.^3^ This finding agrees with our data because we observed higher hematologic complications and increased rates of acute postoperative pain in adults. Additionally, Mack and colleagues,^4^ who, in their study, used a national database, observed higher complication rates in adults when compared with the pediatric population, specifically respiratory and hematologic complications. However, we did not observe increased minor or major complications between the 2 age groups when subgrouping the complications into 3 groups.

The higher complication rate observed in female patients included minor, major, respiratory, and hemorrhagic complications, as well as greater rates of chronic postoperative pain regardless of age. We demonstrated that female patients present at an older age for operative repair, a finding that has been corroborated in other studies.^8^ Age has been shown previously to be strongly associated with complication risk. The rigidity of the chest wall of adults and other acquired medical conditions likely contribute to this risk. Chest wall compliance decreases with age, and this change increases symptom severity; this process could explain the delayed presentation in female patients.^4^

Pain management remains an ongoing challenge in the postoperative setting in patients who have undergone the Nuss procedure. There is a wide variability in postoperative pain scores and chronic pain in the literature.^3^ This is partly the result of the subjectivity of pain scores and assessments, person-to-person variation in pain perception, and differences in postoperative analgesic regimens used. Additionally, the difficulty of the operation and the degree of manipulation of the chest wall may contribute to the differences in pain scores. Multimodal analgesia has been shown to be effective in pain control and in opioid reduction in both adults and pediatric patients.^9^ Furthermore, enhanced recovery after surgery (ERAS) pathways comprise evidence-based interventions that have been used to enhance patients’ recovery postoperatively.^9^ An ERAS protocol focused on the Nuss procedure in adults may be beneficial to the overall outcomes of those patients.

This study has some limitations. This analysis focused on univariate regression analysis as a result of the statistical software used on the TriNetX platform, thereby implying that cofounding factors were not considered. The TriNetX is a retrospective database and may introduce biases, such as selection bias. Given the retrospective design of this study, cause and effect cannot be determined. Because the database used lacked pertinent information on length of stay, mortality, and readmission, we were not able to include any outcomes in the analysis performed. Furthermore, complications that are specific to PE repair such as bar migration were not included in our complication table. This factor could undermine the complication rate forementioned in our study. The database lacked specific information on treating the complications observed; therefore, we were unable to use common surgical complication classification scores such as the Clavien-Dindo classification. Additionally, hospital systems voluntarily provide data to the TriNetX platform. It is possible that centers with better or worse outcomes were not included in the data we analyzed, and this could, in turn, distort our results. Even with these limitations, TriNetX has provided us with an abundance of information given the large sample size it offers. Despite the use of univariate analysis, our data agree with previous institutional and national database analyses.^3^^,^^4^

In conclusion, this study suggests that adults with PE experience significantly increased hemorrhagic complications and greater acute postoperative pain after the Nuss procedure. We also demonstrated a higher minor and major complication rate in female patients regardless of age, including respiratory and hemorrhagic complications, as well as greater rates of chronic postoperative pain.
