# Supplementary figures and images for: Complication Rate of the Nuss Procedure in Adults and Pediatric Patients: National Database Analysis
Source: Ann Thorac Surg Short Rep. 2024 Apr 27;2(3):364–8. doi: 10.1016/j.atssr.2024.04.013 (PMC11708489; doi:10.1016/j.atssr.2024.04.013)

Supplementary Table 1- codes used for patients identification and outcome analysis


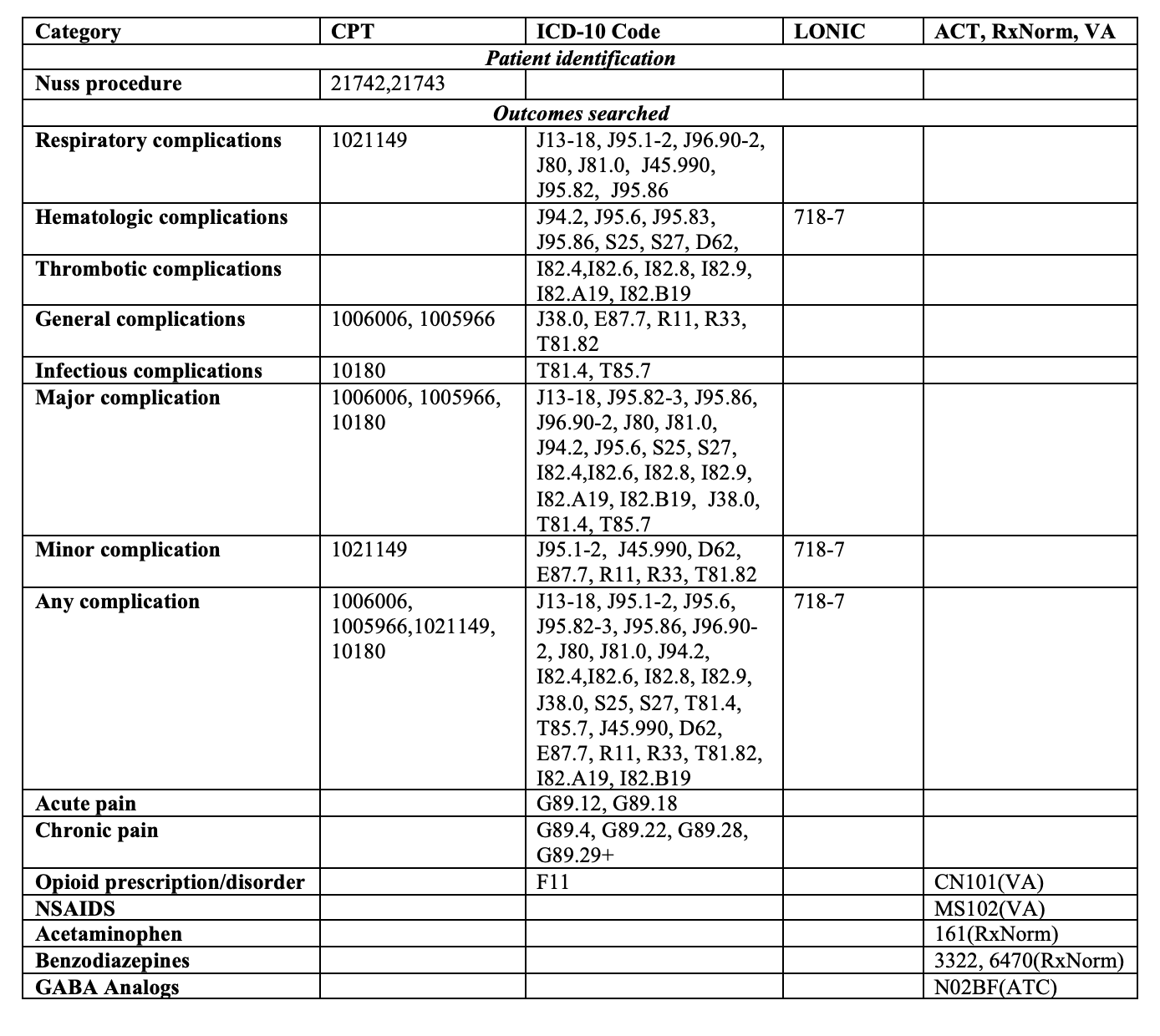

Supplement: Supplemental Table 1 [file mmc1.docx]
