# Supplementary material for: Complication Rate of the Nuss Procedure in Adults and Pediatric Patients: National Database Analysis
Source: Ann Thorac Surg Short Rep. 2024 Apr 27;2(3):364–8. doi: 10.1016/j.atssr.2024.04.013 (PMC11708489; doi:10.1016/j.atssr.2024.04.013)
Supplement: Supplemental Table 2 [file mmc2.docx]

Supplementary Table 2- Categorization of major and minor complicaitons

| Major Complication group | Complication |
| --- | --- |
| Respiratory | Pneumonia  Hypoxic respiratory failure  Hypercapnic respiratory failure  Acute pulmonary edema  Acute respiratory distress syndrome |
| Hematologic | Hemothorax  Hemorrhage in respiratory system |
| Thrombotic | Deep vein thrombosis  Clot, axillary vein  Clot, subclavian vein |
| Other Post-operative | Vocal cord or larynx paralysis |
| Minor Complication group | **Complication** |
| Respiratory | Air leak  Pleural Effusion  Exercise-induced bronchospasm  Pulmonary insufficiency |
| Hematologic | Acute post hemorrhagic anemia |
| Other Post-operative | Fluid overload  Nausea and Vomiting  Urinary Retention  Subcutaneous emphysema  Chronic postoperative pain (>90 days post op) |
